# VitaGraph: building a knowledge graph for biologically relevant learning tasks

**DOI:** 10.1038/s41597-026-07498-4

**Published:** 2026-07-14

**Authors:** Francesco Madeddu, Lucia Testa, Gianluca De Carlo, Michele Pieroni, Andrea Mastropietro, Manuela Petti, Aris Anagnostopoulos, Paolo Tieri, Sergio Barbarossa

**Affiliations:** 1 Department of Computer, Control and Management Engineering, Sapienza University of Rome, 00185 Rome, Italy; 2 Istituto per le Applicazioni del Calcolo, Consiglio Nazionale delle Ricerche, 00185 Rome, Italy; 3 University of Cambridge, Cambridge, UK; 4 Department of Biochemical Sciences, Sapienza University of Rome, 00185 Rome, Italy; 5 Department of Life Science Informatics and Data Science, B-IT, LIMES Program Unit Chemical Biology and Medicinal Chemistry, University of Bonn, Friedrich-Hirzebruch-Allee 6, 53115 Bonn, Germany; 6 Lamarr Institute for Machine Learning and Artificial Intelligence, University of Bonn, Friedrich-Hirzebruch-Allee 6, 53115 Bonn, Germany; 7 Data Science Center, Nara Institute of Science and Technology, 8916-5 Takayama-cho, Ikoma, Nara 630-0192 Japan; 8 Department of Information Engineering, Electronics and Telecommunications, Sapienza University of Rome, 00185 Rome, Italy

## Abstract

The complexity of human biology poses ongoing challenges, driving global interdisciplinary research. Artificial intelligence has become a powerful tool in computational biology, where graph data structures model entities like protein–protein interaction (PPI) networks and gene functional networks. These networks support crucial tasks in network medicine, including gene–disease association prediction, drug repurposing, and polypharmacy side-effect analysis. Reliable machine learning predictions require high-quality data. We present VitaGraph, a comprehensive multi-purpose biological knowledge graph built by integrating and refining multiple public datasets. Extending the Drug Repurposing Knowledge Graph, our pipeline: (a) resolves inconsistencies and redundancies, (b) consolidates information from leading public sources, and (c) enriches graph nodes with expressive features such as molecular fingerprints and gene ontologies. Incorporating biologically and chemically meaningful features enhances machine learning models’ ability to learn accurate, structured embedding spaces. The resulting resource offers a coherent, reliable platform to advance computational biology and precision medicine while enabling benchmarking of graph-based models and offering the opportunity to tackle tasks such as drug repurposing, PPI prediction, and side-effect prediction, among others.

## Background & Summary

Bioinformatics has undergone remarkable advancements in recent years^1^. This field aims to elucidate complex biological phenomena through the analysis of biological data. Of particular interest are omics data^2,3^. The latter encompass molecular information and the interactions among biomolecules across various levels, including genomics, proteomics, and transcriptomics. Notably, the growing interest in understanding protein interactions within organisms has led to the concept of interactome, which involves the construction and analysis of networks representing biological entities and interactions among them. In this context, network medicine^4^ has emerged as a powerful approach for addressing biologically relevant problems by exploiting the structural information encoded in biological networks. One of the most prominent and widely utilized types of such networks is the protein–protein interaction (PPI) network^5^. In these networks, nodes represent genes or proteins, and edges denote physical interactions. PPI networks, such as BioGRID^5^, STRING^6^, and HuRI^7^, have been extensively employed, yielding promising results in various applications, including the identification of gene–disease associations (GDAs)^8–10^ and the prediction of novel protein–protein interactions^11^. Other relevant types of biological networks include gene regulatory networks^12^, in which nodes representing genes are connected based on their involvement in regulating gene expression, ultimately influencing cellular function. These networks provide complementary information to that offered by PPI networks. Gene–disease networks, typically modeled as bipartite graphs, represent associations between genes and diseases; an edge exists if a gene is implicated in the etiology or pathophysiological mechanisms of a given disease. A relevant example of such a dataset is given by DisGeNET^13–15^. Additional relevant types of biomedical networks include drug–drug interaction networks^16^, drug–disease networks^17^, and drug–side-effect networks, exemplified by resources such as SIDER^18^ and OffSides^19^.

Considered individually, these networks, while well-suited to the specific tasks for which they were designed, are insufficient to capture the full complexity of biological systems. Consequently, biological knowledge graphs have been developed with the aim of integrating and harmonizing diverse types of biological information into a unified data resource^20–22^ that can better model the complexity of biological organisms. A notable effort in the field of biomedical knowledge graphs is represented by the Drug Repurposing Knowledge Graph (DRKG)^23^. Originally developed with the aim of identifying potential drug candidates for treating COVID-19^24,25^, DRKG integrates a wide range of biomedical data sources. Due to the breadth and diversity of its integrated information, its applicability could be, in principle, extended beyond drug repurposing to a broad range of biologically relevant tasks that can be framed as link prediction problems within the graph learning paradigm.

Despite its potential, the current version of DRKG suffers from numerous inconsistencies that hinder its practical usability and often result in ambiguous or meaningless outcomes. The most critical issues identified include inconsistent node label formatting, duplication of relationship types, the use of different names to represent semantically and biologically equivalent relationships, and the encoding of pathway-type nodes with non-recoverable identifiers, which severs the connection between node labels and their original biological meaning. Collectively, these inconsistencies introduce substantial noise and redundancy into the graph, artificially inflating the number of nodes and relations without contributing additional information. As a result, downstream models may be biased toward learning spurious dataset artifacts rather than genuine biological signals. In particular, duplicated and overlapping edges increase the likelihood of data leakage between training and evaluation splits, leading to unreliable performance estimates. Furthermore, ambiguous labeling reduces interpretability and hinders meaningful biological validation, thereby limiting the dataset’s practical utility.

Motivated by the potential offered, we adopted DRKG as the foundation for constructing our proposed knowledge graph. Following a careful and comprehensive cleaning process to address these inconsistencies, we enriched the graph with additional information sourced from reliable and domain-specific biomedical databases. Furthermore, we incorporated biochemically meaningful node features to enhance the expressiveness and biological relevance of the embedded knowledge. We thus propose VitaGraph (from the Latin word *vita*, meaning *life*), a novel and versatile knowledge graph tailored for a wide range of biological tasks formulated as link prediction problems, including, but not limited to, drug repurposing, gene–disease association, PPI prediction, and side-effect identification. VitaGraph can also serve as a robust benchmark for assessing link prediction methodologies in the context of network medicine. We also provide a customizable pipeline to generate the proposed dataset, allowing for the inclusion or exclusion of the steps described in the paper, along with code for benchmark purposes. To validate the usability of the knowledge graph, we report an exemplary use case on a drug repurposing task for hypertension treatment.

## Methods

Our dataset is built starting from DRKG, a comprehensive and heterogeneous biological network that integrates diverse biomedical entities and their interactions. DRKG encompasses a wide array of entity types, including genes, compounds, diseases, biological processes, side effects, symptoms, and other biologically relevant concepts. Its primary aim is to facilitate the exploration of disease mechanisms at the molecular level and to support drug repositioning efforts. The graph aggregates data from six major biomedical databases: DrugBank^16,26,27^, Hetionet^28,29^, GNBR^30,31^, STRING^6,32^, IntAct^33,34^, and DGIdb^35–37^, as well as curated information from recent literature, including research related to COVID-19. DrugBank is a richly annotated, comprehensive database containing detailed information about chemical compounds and drug targets (like protein sequences and structures). Hetionet is an integrative biological network containing information about entities such as genes, compounds, and diseases, connected if a given relationship between them exists. GNBR is a network of biomedical relationships obtained through text mining and evaluated against established data sources. STRING is a PPI network, in which physical associations between proteins have been experimentally or computationally determined, or gathered from prior knowledge. IntAct is a database that provides a reliable resource of molecular interactions, curated by several partners of the International Molecular Exchange consortium. Finally, DGIdb is a drug–gene interaction database, and allows the study of the druggability of genes and gene sets.

In its latest release, DRKG comprises 97,238 entities spanning 13 distinct types and contains 5,874,261 triples distributed across 107 edge types. These edge types capture the various relationships that exist among 17 different entity-type pairs, with multiple interaction types possible between the same entity pairs. For example, a compound may interact with a gene both as an inhibitor and as a binder, while gene–gene interactions may include various regulatory and physical associations. This integration of multiple data sources results in a rich, heterogeneous network structure that offers a robust foundation for modeling complex biological systems.

The original DRKG comprises a diverse set of node types, including: anatomy (representing human anatomical structures); ATC (the Anatomical Therapeutic Chemical classification system, used by the World Health Organization to classify drugs); biological process, cellular component, and molecular function (the three branches of the Gene Ontology^38^, which describe gene functions); compound (chemical molecules); disease; gene; pathway (a sequence of molecular events within cells that drive cellular functions); pharmacologic class (categorizing drug compounds based on pharmacological properties); side effect; symptom; and tax (taxonomic identifiers indicating the species origin of genes, such as *homo sapiens*).

**Fig. 1:**
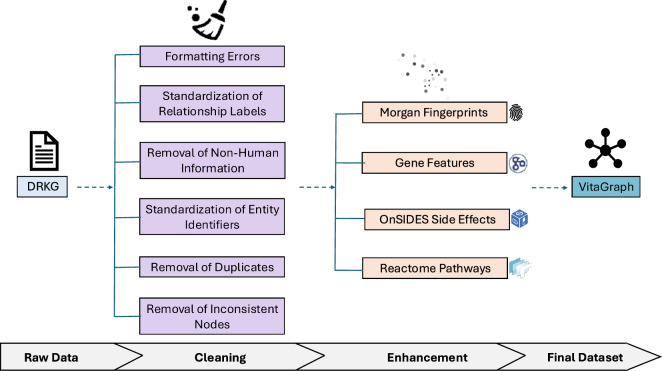
VitaGraph generation pipeline. The schematics shows both the cleaning and the integration with additional features to enhance the expressiveness of the dataset.

Despite its comprehensive scope, DRKG exhibits several shortcomings that limit its usability in downstream tasks. These include the presence of duplicate entries, inconsistent formatting standards, heterogeneous or ambiguous labeling, the inclusion of non-human genes, and invalid or outdated compound identifiers. To address these limitations, a rigorous data cleaning phase was necessary prior to the graph’s integration with additional data sources and the enrichment of nodes with biologically and chemically meaningful features. An illustration of the complete dataset generation pipeline is given in Fig. 1.

### Filtering triples with formatting errors

The dataset was analyzed for formatting inconsistencies that could adversely affect downstream processing and analysis. The first issue identified concerned the representation of entity identifiers. While the majority of entries adhered to the format entity_type::database_source:entity_id, a subset lacked the database source. For instance, in DRKG, the node Compound::pubchem:17753879 is in the correct format while the node Compound::DB01116 is missing the database source, requiring user efforts to understand that the identifier is a DrugBank ID. To ensure consistency and facilitate the traceability of entity origins, all identifiers were standardized to follow the former format, explicitly indicating the source database.

Second, node labels containing the semicolon character (“;”) were flagged, as this character often suggested that multiple entities had been erroneously merged into a single node, like, for instance, the node label Gene::108068;108069;14800;53623. These inconsistent entries were removed, resulting in the elimination of 1,122 triples.

Third, the dataset included node labels in which compounds were separated by the pipe character (“|”). For example, the node label Compound::MESH:C499810|MESH:C040810. These cases were interpreted as potential formatting errors or ambiguous representations of compound combinations. Due to the uncertainty surrounding their intended semantics and their limited prevalence, these entries were also excluded from the dataset, resulting in the elimination of 98 triples.

### Standardization of relationship labels

As previously noted, the original DRKG comprises 107 distinct edge types (interaction types), many of which are expressed using synonymous terms or alternative codes. To reduce redundancy and enhance semantic consistency across the dataset, we performed a harmonization step in which synonymous interaction labels were mapped to a set of unified and standardized terms. This mapping facilitates more coherent interpretation and analysis of the graph’s relational structure. In Table 1, we report the mapping between the interaction types in VitaGraph and the original sources.

**Table 1:** Mapping of interaction types to their corresponding database sources.

New interaction	DrugBank	GNBR	Hetionet	STRING	IntAct	DGIdb	bioRxiv
Activator	—	A +	—	—	—	Activator, Agonist	—
Blocker	—	A-	—	—	—	Antagonist, Blocker, Channel Blocker	—
CMP_BIND	Target	B	CbG	—	Direct Interaction, Association, Physical Interaction	Binder	DHG*
ENZYME	Enzyme	Z	—	—	—	—	—
EXPRESSION	—	E	—	Expression	—	—	—
Regulation	—	Rg	Gr>G	—	—	—	—
UPREGULATION	—	E+	CuG	—	—	—	—
DOWNREGULATION	—	E-, N	CdG	—	—	Inhibitor	—
GENE_BIND	—	B	GiG	Binding	Physical Assoc., Assoc., Dir. Int.	—	HGHG**
TREATMENT	Treats	T	CtD	—	—	—	—
J_c	—	J	—	—	—	—	—
J_g	—	J	—	—	—	—	—
gene_OTHER_cmp	—	—	—	—	—	Other	—
gene_OTHER_gene	—	—	—	Other	—	—	—

Each row corresponds to a unified relation type, while the columns indicate the presence or absence of that relation in the original source databases, with the corresponding interaction name if that interaction is present. For example, the edge types labeled “Activator” and “Agonist” from the DGIdb dataset are consolidated into a single standardized Activator relation type in our dataset. A dash (“–”) denotes that the corresponding interaction type is not present in the given data source (*DrugHumGen, **HumGenHumGen).

### Removal of non-human information

To focus the dataset on human biology and eliminate biases from COVID-19-related studies, virus-related relationships were excluded. Non-human genes and their interactions were also removed to avoid introducing noise into human-specific analyses. This filtering step, aimed at enhancing semantic consistency and biological relevance, led to the removal of 135,294 triples and 17,553 non-human genes. For broader research interests, the pipeline allows users to retain non-human interactions by disabling this cleaning step.

### Standardization of entity identifiers

The original DRKG was constructed by merging multiple databases that lacked a unified entity identification system, resulting in entities being represented by multiple identifiers. This redundancy led to information loss and fragmentation. To address this issue, we implemented a systematic approach to unify entity identifiers by leveraging additional data sources. Mapping entity identifiers poses considerable challenges, particularly because complete cross-database correspondence cannot be ensured, as certain entities may exist exclusively within specific datasets.

#### Compound identifier mapping

For the standardization of compound identifiers, we employed the UniChem platform^39^, which offers comprehensive cross-referencing services by aggregating information from databases such as ChEMBL^40–42^ and ChEBI^43–45^. This approach enabled consistent compound mappings across datasets, with PubChem identifiers adopted as the preferred standard due to their extensive coverage^46–48^. Through this standardization process, 2,508 redundant compound IDs were identified and resolved.

#### Disease mapping

For disease entity standardization, we leveraged the Human Disease Ontology database^49,50^, which provides comprehensive cross-referencing between Disease Ontology (DOID)^51^, Medical Subject Headings (MeSH)^52^, and Online Mendelian Inheritance in Man (OMIM) identifier systems^53^. Through this standardization process, 118 redundant disease IDs were identified and resolved.

#### Gene mapping

Genes are identified using the NCBI ID. However, genes retrieved from the DrugBank database may not have a corresponding NCBI ID. To preserve the information carried by such genes, we retained the DrugBank ID for 62 genes for which no mapping was possible.

### Removal of duplicates

The steps described above aim to map the largest possible number of entities and interactions to a standardized space, allowing the identification of redundant information. To ensure data consistency, we checked for duplicate edges. In the first stage, exact duplicate triples are identified and eliminated. However, due to the possibility that certain relationships are recorded with the head and tail nodes in reversed order, an additional step is required. Taking this into account, duplicate entries are detected and removed. The de-duplication procedure resulted in the elimination of 842,262 duplicate triples, thereby significantly improving the structural integrity and consistency of the graph.

### Additional cleaning procedures

Pathway nodes presented several inconsistencies across the various data sources used. Therefore, we initially removed all pathway nodes and their associated connections from the graph. In *Pathways*, we will describe the inconsistencies and how this information was integrated back into the knowledge graph in a coherent manner. Finally, as an additional cleaning step, nodes representing Taxonomy were removed, as they were linked to only a single entity and thus did not contribute additional meaningful information to the knowledge graph. The output of the proposed cleaning pipeline is a refined knowledge graph, free from redundancy, noise, and ambiguous informational content. This intermediate representation serves as a foundation for the construction of VitaGraph, incorporating node features derived and systematically processed from additional data sources, as described in the next section.

### Enlarging the scope: toward VitaGraph

A hallmark of our dataset lies in its integration of additional data sources as well as the definition of biologically meaningful and expressive features. On the one hand, we augment the information content by adding pathway and drug–side-effect information from new databases. On the other hand, we enrich the graph nodes with features. Drawing from both biological and chemical domains, the nodes within the graph are endowed with a comprehensive set of descriptors that capture their biochemical significance and role in the biological knowledge. This design paradigm ensures that, beyond the relational structure of the graph, the intrinsic properties of the individual entities contribute substantively to graph learning processes for biological tasks. The addition of node features is essential for a coherent learning of graph neural network-based models. Node features provide information that is propagated throughout the network via edges. Given that graph topology alone may not be sufficient to solve specific tasks, features enable nodes to be clearly distinguished by graph learning models, leading to the generation of expressive node embeddings that take into account not only connectivity patterns but also the specific information carried by each node. These considerations are consistent with prior work showing that effective learning on graphs typically requires combining structural information with node attributes^54,55^. This enriches the graph structure, leading to VitaGraph, a new, more comprehensive, and expressive knowledge graph for life science-centered machine learning and network science.

#### Pathways

As mentioned in *Additional cleaning procedures*, all 1,822 pathway nodes were removed due to inconsistencies. Specifically, pathway identifiers in DRKG (inherited from Hetionet) utilize internal surrogate keys rather than stable, external database identifiers. This choice severs the direct link between a node and its annotations in source databases. For example, the DRKG pathway identifier PC7_6941 is a local key that yields no matches in the Pathway Commons database^56^, effectively rendering the node “orphaned” for downstream enrichment analysis. To compensate for this, we chose to rely on a single, widely recognized and adopted database: Reactome^57–59^. Subsequently, we introduced 2,153 new pathway nodes into the graph and connected them to the genes involved in those pathways with 68,380 edges, as detailed in the Reactome database. This resulted in more extensive coverage and a more consistent and unambiguous integration of molecular pathway knowledge within the graph.

#### Drug–side-effect information

Given the relevance of predicting the side effects of a compound, we integrated the OnSIDES dataset^60,61^ for compound–side-effect edges. OnSIDES, curated from real-world pharmacovigilance data, offers a rich source of high-confidence associations that complement existing resources. By incorporating it, we aim to broaden the coverage of known adverse drug reactions, thereby supporting downstream research and analysis with a more comprehensive foundation of pharmacological knowledge. We incorporated the latest version of the dataset (v3.0.0) and selected only the highest-confidence set of edges. To ensure data consistency and avoid introducing noise, we included only those edges involving compounds already present in our graph. Additionally, we mapped and standardized compound and side effect identifiers to prevent redundancy. The mapping of side effect identifiers was accomplished leveraging the SIDER database that contains mappings of MedDRA IDs to our target identifiers. As a result, a total of 339,867 edges were integrated into the dataset.

#### Elimination of compounds without SMILES representations

Accurate chemical representation is essential for computational analyses involving molecular compounds. SMILES (Simplified Molecular Input Line Entry System) strings^62^ offer a standardized format for describing chemical structures. At this stage, the dataset was cross-referenced with a comprehensive compound dictionary that incorporates SMILES annotations curated from DrugBank, GNBR, Hetionet, IntAct, DGIdb, and PubChem. Entries corresponding to compounds lacking an associated SMILES representation were excluded. Such compounds are typically either no longer available (e.g., withdrawn from the market) or proprietary in nature, with undisclosed chemical structures. Consequently, these entries were removed to maintain data openness and research reproducibility. Moreover, SMILES representations are necessary for subsequent processing steps, such as the generation of Morgan fingerprints (see *Morgan fingerprint generation*), and this led to retaining only those compounds suitable for structural analysis. This filtering process eliminated 3,872 compounds and 239,219 edges.

#### Morgan fingerprint generation

To further augment the chemical information content of the knowledge graph, compound nodes were associated with their corresponding Morgan fingerprints^63,64^. Morgan fingerprints are a type of circular fingerprint that captures the local structural features of a molecule. By iteratively examining atomic neighborhoods within a defined radius, these fingerprints translate substructural characteristics into fixed-length binary vectors, where each bit denotes the presence (1) or absence (0) of a particular molecular feature. This representation is particularly valuable for similarity searches and machine learning applications in chemoinformatics. Each compound node was assigned a fingerprint feature vector of length 2,048, as is common in the field for machine representation of chemical entities^65^. We successfully generated and appended 15,831 fingerprints, thereby improving the chemical characterization within the graph.

#### Gene features

**Fig. 2:**
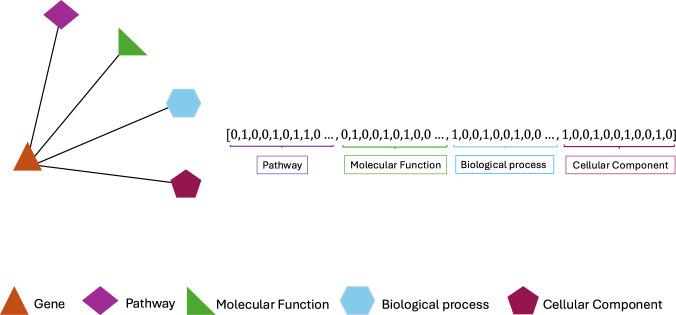
Construction of the gene feature vector. A one-valued entry in the feature vector indicates that the gene was connected to a given entity of that type in DRKG.

**Fig. 3:**
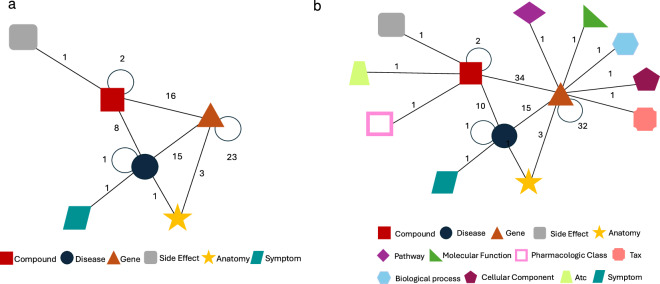
Comparison of the new VitaGraph (**a**) and the DRKG schema (**b**). In VitaGraph, only a limited number of node and edge types are preserved, augmenting the coherence and reducing the noise within the graph.

To incorporate rich biological context, we enhanced the gene nodes by encoding functional information into binary feature vectors. These vectors capture associations with biological pathways, molecular functions, biological processes, and cellular components. In DRKG, such associations were originally represented as edges connecting gene nodes to nodes corresponding to these biological entities. Because nodes representing such entities were linked exclusively to gene nodes and lacked self-loops (thus forming star-shaped subgraphs), we opted to collapse these structures and embed the associated information as gene node features. This approach reduced the graph’s complexity while simultaneously enhancing the expressive capacity of gene nodes. Specifically, 2,153 pathways were encoded using binary feature vectors and incorporated into the gene feature vectors. The same procedure was applied for 2,884 molecular functions, 11,381 biological processes, and 1,391 cellular components. The resulting feature vectors ensure that each gene is accompanied by a detailed functional profile. Although it does not explicitly model the full directed acyclic graph structure of the Gene Ontology hierarchy, this representation is still able to capture hierarchical signals without preserving nodes or introducing additional edges, thus reducing graph complexity. Fig. 2 illustrates how such a feature vector is derived from the original graph topology: edges that previously existed in DRKG are now represented as one-valued entries in the encoded vector. Fig. 3 showcases the structure of VitaGraph compared with the DRKG schema.

## Data Records

VitaGraph^66^ is freely hosted on Kaggle, and it is available at the following link: 10.34740/kaggle/ds/7415432. The dataset is distributed as a tab-separated file vitagraph.tsv, as a turtle file vitagraph.ttl, and as an N-triples file vitagraph.nt, to maximize compatibility. Additionally, we provide several auxiliary files that contain node features and additional information used to build the dataset. More precisely, the repository contains the following files:

- vitagraph.tsv: contains all the triples (head, interaction, tail), along with two columns source and type, which specify the source dataset of the interaction and its type (e.g., Compound–Gene)
- vitagraph.ttl: contains all the triples in turtle format
- vitagraph.nt: contains all the triples in N-triples format
- Folder auxiliary: contains the files necessary to generate VitaGraph
  - all_cmp_info.csv: information for each compound in the dataset (e.g., SMILES strings, compound names)
  - compounds_mapping.csv: mapping of compounds across datasets
  - disease_mapping.csv: mapping of diseases across datasets
  - drugbank_to_ncbi_gene_id.csv: mapping of DrugBank genes to NCBI genes
  - genes_to_remove.csv: list of non-human genes
  - onsides.csv: edges from OnSIDES (compound–side-effect)
  - reactome.csv: edges from Reactome (gene–pathway)
- Folder drkg: contains the original DRKG dataset
  - drkg.tsv: edges of the DRKG dataset
- Folder vitagraph_features: contains VitaGraph edges, features, and feature-mapping files
  - BiologicalProcess_feature_vector_position_dict.json: mapping of each biological process to its relative index position in the gene feature vector
  - CellularComponent_feature_vector_position_dict.json: mapping of each cellular component to its relative index position in the gene feature vector
  - MolecularFunction_feature_vector_position_dict.json: mapping of each molecular function to its relative index position in the gene feature vector
  - Pathway_feature_vector_position_dict.json: mapping of each pathway to its relative index position in the gene feature vector
  - comp_features.csv: Morgan fingerprints for each compound
  - gene_features.csv: binarized features for each gene
- Folder vitagraph_no_features: contains the vitagraph_no_features dataset
  - vitagraph_no_features.tsv: contains the edges of the vitagraph_no_features dataset, the dataset version without node features

### Nodes

Node entities in VitaGraph encompass a wide range of biochemical concepts. A detailed description of the entity types, their meanings, data sources of origin, and how they are identified in the knowledge graph is provided in Table 2.

**Table 2:** Node entity types in VitaGraph with descriptions, origin datasets, and identifier formats.

Entity type	Description	Origin datasets	Identifier format example
**Gene**	DNA segments that encode functional products (primarily proteins), essential for understanding molecular functions and genetic interactions.	NCBI, DrugBank	Gene::NCBI:1257 Gene::drugbank:BE0004351
**Compound**	Small molecules or drugs of therapeutic interest, cross-referenced across multiple chemical repositories.	ChEMBL, PubChem, MolPort, ZINC, ChEBI	Compound::CHEMBL:CHEMBL71920 Compound::PubChem_Compounds:447484 Compound::molport:MolPort-006-169-233 Compound::zinc:ZINC000031417519 Compound::CHEBI:29995
**Disease**	Pathological processes characterized by signs/symptoms, with known or unknown etiology, pathology, and prognosis.	MeSH, DOID, bioRxiv (SARS-CoV2-related), OMIM	Disease::MESH:D004715 Disease::DOID:3121 Disease::bioRxiv:SARS-CoV2 nsp15 Disease::OMIM:138800
**Anatomy**	Anatomical structures, annotated with Uberon codes, an integrated cross-species anatomical ontology^71^.	Uberon	Anatomy::UBERON:0000995
**Side effect**	Adverse reactions or undesirable outcomes associated with drugs or medical interventions, annotated according to the Unified Medical Language System^72^.	UMLS	SideEffect::umls:C0155616
**Symptom**	Clinical manifestations or observable signs associated with diseases or pathological conditions.	MeSH	Symptom::MESH:D015878

### Edges

In VitaGraph, edge types are organized into main categories and corresponding subcategories. For each main category, the associated subtypes are listed alongside brief descriptions of the relationships they represent. Each subtype is prefixed with the name of its source (e.g., DGIDB, DRUGBANK, GNBR, Hetionet, INTACT, STRING, or bioRxiv) to clearly indicate both the origin and the nature of the relationship. Details can be found in Table 3.

**Table 3:** Edge (relation) types in VitaGraph with source datasets and descriptions.

Edge type	Relation (dataset)	Description
**Anatomy–Gene**	AdG, AeG, AuG (Hetionet)	Associations between anatomy and genes: curated links, expression/localization data, nuanced curated relations.
**Compound–Compound**	ddi-interactor-in (DrugBank); CrC (Hetionet)	Drug–drug interactions or compound similarity (chemical resemblance, shared mechanisms).
**Compound–Disease**	C, J_c, Mp, Pa, Pr, Sa (GNBR); TREATMENT (DrugBank, GNBR, Hetionet); CpD (Hetionet)	Compound–disease links: inhibits growth, pathogenesis role, biomarkers, alleviates/reduces, prevents/suppresses, adverse events, treatment or palliation.
**Compound–Gene**	carrier, ENZYME, CMP_BIND (multi-source); ACTIVATOR, BLOCKER, UPREGULATION, DOWNREGULATION, E, K, O (GNBR/DGIDB/Hetionet); ALLOSTERIC_MODULATOR, ANTIBODY, MODULATOR, PARTIAL_AGONIST, POSITIVE_ALLOSTERIC_MODULATOR, gene_OTHER_cmp (DGIDB)	Compound–gene interactions: carrier or enzyme targeting; binding (orthosteric/allosteric); activation/blocking; up-/downregulation; metabolism/transport; antibody binding; modulators (direct or positive allosteric); partial agonism; undefined categories.
**Compound–SideEffect**	CcSE (Hetionet–OnSIDES)	Associates a compound with a side effect.
**Disease–Anatomy**	DlA (Hetionet)	Disease manifests in or is associated with an anatomical structure.
**Disease–Disease**	DrD (Hetionet)	Links diseases by comorbidity or shared pathological mechanisms.
**Disease–Gene**	DaG, DdG, DuG (Hetionet); Coronavirus_ass_host_gene, Covid2_acc_host_gene (bioRxiv); D, G, J_g, L, Md, Te, U, Ud, X, Y (GNBR)	Disease–gene associations: curated (up-/downregulation), coronavirus host links, causal mutations/polymorphisms, biomarkers, improper regulation, therapeutic effects, progression roles.
**Disease–Symptom**	DpS (Hetionet)	Disease linked to one of its characteristic symptoms.
**Gene–Gene**	ACTIVATION, EXPRESSION, INHIBITION, REGULATION, REACTION, PTMOD (STRING/GNBR/Hetionet); GENE_BIND (multi-source); GcG (Hetionet); E+ , V+ , W, H, I, Q (GNBR); ADP_RIBOSYLATION_REACTION, CLEAVAGE_REACTION, DEPHOSPHORYLATION_REACTION, PHOSPHORYLATION_REACTION, PROTEIN_CLEAVAGE, UBIQUITINATION_REACTION, COLOCALIZATION (IntAct); gene_OTHER_gene (STRING)	Gene–gene relations: physical binding, coexpression, regulation, activation/inhibition, post-translational modifications, enzymatic reactions (cleavage, phosphorylation, ubiquitination, ribosylation), co-localization, pathway involvement, functional enhancement.

## Data Overview

VitaGraph is composed of 48,058 nodes and 4,004,583 edges. In Tables 4 and 5, we illustrate details on the data sources for nodes and edges. Table 6 provides a summary of the main statistics for VitaGraph in comparison to DRKG. The refined and optimized VitaGraph can be used for advanced computational analyses, machine learning, and data mining in bioinformatics and network medicine tasks.

**Table 4:** Node type to source.

Source	Anatomy	Compound	Disease	Gene	SideEffect	Symptom	Total
CHEBI	0	176	0	0	0	0	176
CHEMBL	0	77	0	0	0	0	77
DOID	0	0	2 390	0	0	0	2 390
MESH	0	0	2 356	0	0	415	2 771
NCBI	0	0	0	20 844	0	0	20 844
OMIM	0	0	31	0	0	0	31
PubChem_Compounds	0	15 302	0	0	0	0	15 302
UBERON	400	0	0	0	0	0	400
bioRxiv	0	0	27	0	0	0	27
drugbank	0	0	0	62	0	0	62
molport	0	221	0	0	0	0	221
umls	0	0	0	0	5 704	0	5 704
zinc	0	53	0	0	0	0	53
**Total**	400	15 829	4 804	20 906	5 704	415	48 058

**Table 5:** Edge types with their respective sources.

Relation	DGIDb	DrugBank	GNBR	Hetionet	IntAct	OnSIDES	STRING	bioRxiv	Total
Anatomy-Gene	0	0	0	726 156	0	0	0	0	726 156
Compound-Compound	0	1 161 176	0	6 441	0	0	0	0	1 167 617
Compound-Disease	0	4 502	68 455	609	0	0	0	0	73 566
Compound-Gene	22 750	8 671	34 469	41 751	1 563	0	0	24 248	133 452
Compound-SideEffect	0	0	0	138 017	0	284 235	0	0	422 252
Disease-Anatomy	0	0	0	3 602	0	0	0	0	3 602
Disease-Disease	0	0	0	543	0	0	0	0	543
Disease-Gene	0	0	65 679	27 936	0	0	0	458	94 073
Disease-Symptom	0	0	0	3 357	0	0	0	0	3 357
Gene-Gene	0	0	23 994	461 263	141 926	0	723 259	29 523	1 379 965
**Total**	22 750	1 174 349	192 597	1 409 675	143 489	284 235	723 259	54 229	4 004 583

**Table 6:** Comparison between VitaGraph and DRKG.

Dataset	Triples	Nodes	Relationship types	Node types
DRKG	5 874 261	97 238	99	13
VitaGraph	4 004 583	48 058	71	6
**Difference**	1 869 678	49 180	28	7

Pathways, cellular components, molecular functions, and biological processes are included as node features in VitaGraph.

## Technical Validation

To validate the use and utility of the proposed dataset, we employed three baseline models capable of learning from multi-relational and heterogeneous graph structures. Specifically, we conducted experiments on three prominent link prediction tasks commonly studied in the bioinformatics and network medicine domains, namely drug repurposing, PPI prediction, and drug–side-effect detection, modeled as multi-relational link prediction problems, where the objective is not only to determine whether two nodes are connected, but also to predict the type of interaction between them. These tasks serve as strong benchmarks for assessing the utility and generalizability of knowledge graph-based representations in biomedical applications.

To rigorously assess the impact of our dataset enhancements, we performed experiments across three distinct versions of the dataset: (1) the original DRKG dataset, which serves as a baseline, (2) a cleaned version of DRKG including the Reactome and OnSIDES merge without the addition of the features presented in *Enlarging the scope: toward VitaGraph*, and (3) the final VitaGraph, which includes both the cleaned structure and node features. This comparative setup enables us to isolate and quantify the contributions of data cleaning and feature enrichment to the models’ predictive performance on exemplary tasks.

We employed modified versions of the relational graph convolutional network (R-GCN)^67^, relational graph attention network (R-GAT)^68^, and composition-based multi-relational graph convolutional networks (CompGCN)^69^, which were adapted to learn from heterogeneous multi-relational graph data. These architectures can leverage the complex relational structure of biomedical knowledge graphs, such as ours. Moreover, their flexibility in handling heterogeneous data enables them to learn from nodes that possess varying feature sets.

To train the model on different tasks, specific subsets of triples were selected. For the PPI task, we used edges connecting pairs of gene entities. For the drug repurposing task, we used edges between compound and gene entities. Finally, for the side effect prediction task, we used edges linking compounds to side effects. While the loss is computed only on these task-specific subsets, all other edges remain in the graph to support message passing and information flow. We adopted a split of 70% training, 10% validation, and 20% test based on the target triples for each task. Notably, this experimental setup was devised solely to show possible effective use cases for VitaGraph.

Tables 7, 8, 9 report the performance of the models across the various dataset versions and task configurations.

**Table 7:** Drug repurposing use case.

Dataset	Model	AUROC	AUPRC	MRR	Hits@1	Hits@3	Hits@10
DRKG	R-GCN	**0.914 ± 0.030**	**0.896 ± 0.028**	0.148 ± 0.035	0.000 ± 0.000	0.025 ± 0.075	**0.650 ± 0.166**
	R-GAT	0.876 ± 0.112	0.871 ± 0.113	0.133 ± 0.069	0.025 ± 0.075	0.025 ± 0.075	0.550 ± 0.350
	CompGCN	0.904 ± 0.023	0.881 ± 0.028	**0.246 ± 0.093**	**0.075 ± 0.115**	**0.350 ± 0.166**	0.550 ± 0.187
VitaGraph (no feat)	R-GCN	**0.928 ± 0.004**	**0.917 ± 0.007**	0.176 ± 0.090	0.000 ± 0.000	**0.175 ± 0.195**	0.475 ± 0.305
	R-GAT	0.858 ± 0.076	0.842 ± 0.079	**0.239 ± 0.267**	**0.125 ± 0.301**	0.175 ± 0.297	0.575 ± 0.297
	CompGCN	0.827 ± 0.077	0.796 ± 0.067	0.209 ± 0.121	0.050 ± 0.150	0.150 ± 0.166	**0.675 ± 0.225**
VitaGraph	R-GCN	0.887 ± 0.060	0.870 ± 0.053	0.219 ± 0.126	0.050 ± 0.100	0.250 ± 0.354	0.825 ± 0.160
	R-GAT	**0.937 ± 0.002**	**0.929 ± 0.004**	0.189 ± 0.177	**0.100 ± 0.200**	0.100 ± 0.200	0.400 ± 0.250
	CompGCN	0.923 ± 0.013	0.901 ± 0.016	**0.257 ± 0.046**	0.050 ± 0.100	**0.400 ± 0.122**	**0.925 ± 0.160**

The results are presented as mean and standard deviation over 10 independent runs.

**Table 8:** Compound–side-effect use case.

Dataset	Model	AUROC	AUPRC	MRR	Hits@1	Hits@3	Hits@10
DRKG	R-GCN	**0.881 ± 0.024**	**0.839 ± 0.034**	0.126 ± 0.136	0.050 ± 0.150	0.050 ± 0.150	0.300 ± 0.187
	R-GAT	0.811 ± 0.085	0.772 ± 0.086	**0.225 ± 0.203**	0.099 ± 0.229	0.099 ± 0.229	**0.649 ± 0.320**
	CompGCN	0.870 ± 0.025	0.829 ± 0.025	0.158 ± 0.144	0.050 ± 0.150	**0.150 ± 0.200**	0.300 ± 0.384
VitaGraph (no feat)	R-GCN	**0.899 ± 0.021**	**0.846 ± 0.024**	**0.358 ± 0.222**	**0.250 ± 0.250**	**0.325 ± 0.251**	0.500 ± 0.316
	R-GAT	0.840 ± 0.098	0.784 ± 0.089	0.116 ± 0.057	0.000 ± 0.000	0.075 ± 0.225	**0.575 ± 0.371**
	CompGCN	0.865 ± 0.027	0.807 ± 0.025	0.150 ± 0.112	0.000 ± 0.000	0.200 ± 0.350	0.350 ± 0.339
VitaGraph	R-GCN	0.888 ± 0.019	0.827 ± 0.023	**0.326 ± 0.190**	**0.200 ± 0.245**	0.325 ± 0.225	0.475 ± 0.261
	R-GAT	**0.891 ± 0.005**	**0.828 ± 0.008**	0.276 ± 0.066	0.000 ± 0.000	**0.475 ± 0.207**	**0.825 ± 0.195**
	CompGCN	0.824 ± 0.052	0.776 ± 0.043	0.134 ± 0.069	0.000 ± 0.000	0.125 ± 0.168	0.450 ± 0.245

The results are presented as mean and standard deviation over 10 independent runs.

**Table 9:** Protein-protein interaction use case.

Dataset	Model	AUROC	AUPRC	MRR	Hits@1	Hits@3	Hits@10
DRKG	R-GCN	0.885 ± 0.032	0.871 ± 0.029	0.157 ± 0.085	0.050 ± 0.100	0.100 ± 0.122	0.500 ± 0.274
	R-GAT	0.904 ± 0.068	0.886 ± 0.081	**0.348 ± 0.269**	**0.225 ± 0.325**	**0.275 ± 0.325**	**0.725 ± 0.207**
	CompGCN	**0.924 ± 0.038**	**0.914 ± 0.041**	0.245 ± 0.263	0.125 ± 0.301	**0.275 ± 0.325**	0.600 ± 0.300
VitaGraph (no feat)	R-GCN	**0.893 ± 0.074**	**0.890 ± 0.069**	**0.280 ± 0.204**	**0.175 ± 0.225**	**0.275 ± 0.261**	**0.575 ± 0.354**
	R-GAT	0.875 ± 0.115	0.865 ± 0.115	0.121 ± 0.070	0.025 ± 0.075	0.025 ± 0.075	0.325 ± 0.275
	CompGCN	0.859 ± 0.041	0.845 ± 0.039	0.150 ± 0.055	0.000 ± 0.000	0.100 ± 0.200	0.475 ± 0.261
VitaGraph	R-GCN	0.859 ± 0.059	0.852 ± 0.054	0.139 ± 0.069	0.025 ± 0.075	0.125 ± 0.168	0.475 ± 0.325
	R-GAT	**0.896 ± 0.036**	**0.893 ± 0.034**	**0.337 ± 0.315**	**0.275 ± 0.343**	**0.275 ± 0.343**	**0.525 ± 0.378**
	CompGCN	0.884 ± 0.061	0.882 ± 0.059	0.180 ± 0.141	0.075 ± 0.160	0.075 ± 0.160	0.475 ± 0.284

The results are presented as mean and standard deviation over 10 independent runs.

At first glance, the results on the original DRKG appear promising. However, upon deeper inspection, we notice that these results are significantly affected by data leakage between the training, validation, and test sets, further motivating the introduction of VitaGraph. To identify potential leakage, we examined the three dataset splits by checking for overlap in three ways: 1) duplicate identification, 2) relation-level redundancy, and 3) entity-level redundancy. With the first approach, we searched for identical triples across splits, including their inverse forms (i.e., A interacts with B in the training set, and B interacts with A in the test set). This directly indicates leakage of connectivity information. In the second, we applied the pipeline’s relation standardization across the splits and checked for overlaps. In DRKG, the same semantic interaction can be represented by multiple redundant relation IDs. Although these IDs are different, they retain the same structural information. As a result, even if a relation appears with a different name in the test set, the model may still recognize and exploit its topology learned from the training set. By standardizing relations, we can identify overlapping interactions that reveal this type of data leakage. Finally, for entity-level redundancy, we standardized entities across splits to detect duplicate nodes, as the same entity may be duplicated under different IDs. These redundant entities typically share similar latent representations and have overlapping neighborhoods. We consider it a form of leakage when an edge is masked for a node in the test set, but the same edge is unmasked for a redundant node in the training set. In this scenario, the model can infer the missing edge based on the connections of a nearly identical redundant entity’s latent representation. Table 10 quantifies the extent of data leakage across tasks, measured as the ratio of tuples that are present in both training and validation sets (or training and test sets). We found substantial leakage in the PPI task and the drug repurposing task, significantly compromising the reliability of the results. In contrast, the side-effect prediction task remains unaffected. This is because its edges, which connect compounds to side effects, all originate from the same source dataset (SIDER) and are not subject to redundancy. Therefore, the performances on the original DRKG can be mostly attributed to redundancies and data leakage.

**Table 10:** Leakage ratios for train/val and train/test splits, measured as mean and standard deviation over ten independent runs with different random splits.

Split	PPI	Drug repurposing	Compound–side-effect
Train/Val	0.655 ± 0.002	0.182 ± 0.003	—
Train/Test	0.655 ± 0.001	0.187 ± 0.002	—

No leakage is present for the compound–side-effect task.

This validation demonstrates the potential of graph learning tasks using VitaGraph, motivating the development of more advanced learning models that can lead to novel, biologically relevant discoveries.

### Latent space analysis

**Fig. 4:**
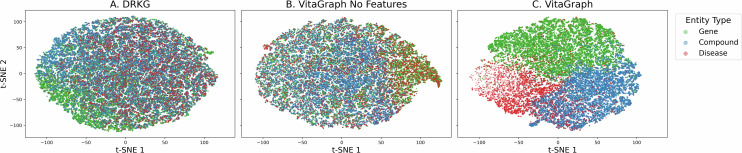
Latent space analysis. Visual representation and comparison of the produced embeddings across the three datasets DRKG (**A**), VitaGraph no features (**B**), and VitaGraph (**C**), produced with t-SNE^70^.

We evaluate the impact of our cleaning pipeline and the added features by examining the quality of embeddings across different dataset versions. Table 11 presents the results obtained with the R-GAT model through several clustering metrics, and Fig. 4 complements this with a visual comparison of the embeddings projected into a 2D latent space.

**Table 11:** Comparison of VitaGraph, VitaGraph without features, and DRKG across multiple clustering metrics.

Metrics	VitaGraph	VitaGraph (no features)	DRKG
↓ Davies–Bouldin	**2.593**	6.506	47.325
↑ kNN Accuracy	**0.861**	0.353	0.350
↑ kNN Acc. (Selected)	**0.966**	0.590	0.570
↑ Purity	**0.717**	0.334	0.428
↑ Purity (Selected)	**0.726**	0.504	0.610
↑ Silhouette	**0.009**	−0.125	−0.101
↑ Silhouette (Selected)	**0.063**	−0.119	0.001

Bold values indicate the best performance per metric. (Selected) refers to a subset of nodes related to the drug repurposing task (genes, diseases, and compounds).

The results show that the added features enable the construction of a more discriminative and semantically meaningful embedding space. To ensure that the analysis focuses on the most relevant regions of the graph for downstream tasks, we restrict attention to disease, gene, and compound nodes, which undergo significant structural changes and play a central role in practical applications.

Fig. 4 highlights these differences across datasets. In DRKG and VitaGraph without features, the embeddings divided by node type show little separation in the latent space. By contrast, in VitaGraph with features, the embeddings form clear and well-separated clusters. Interestingly, within these clusters we can also observe meaningful substructures, such as genes grouped by similar biological functions or compounds associated with similar effects.

### Use case: drug repurposing for hypertension treatment

As a complementary example to the technical benchmarking reported above, we provide here an illustrative downstream application of VitaGraph to support a realistic hypothesis-generation workflow for drug repurposing. In particular, we focus on hypertension as a representative complex trait, and we show how the graph can be used to prioritize candidate compounds whose predicted molecular interactions are mechanistically coherent with hypertension-related biology. Importantly, this use case is intended to exemplify how VitaGraph can be operationally employed in an applied setting; it does not constitute clinical validation, and any candidate identified through this pipeline would require dedicated experimental or clinical assessment.

#### Downstream pipeline

The proposed workflow follows a standard knowledge graph completion paradigm, leveraging the same family of multi-relational GNN models adopted in the technical validation (e.g., R-GCN, CompGCN, R-GAT) and using the full heterogeneous graph to enable message passing across entity types. Concretely, the pipeline consists of the following steps:

- **Model training for inference:** A link prediction model (R-GAT) is trained on the complete set of available graph edges (i.e., without holding out validation/test triples) to maximize the amount of information available for inference. In this setting, the goal is not to estimate generalization metrics, but to obtain a scoring function for ranking candidate interactions.
- **Disease and gene set definition:** A target disease node is selected among the disease entities present in the dataset (Disease::<OriginDataset>:<diseaseid>). For hypertension, we retrieve the set of disease-associated genes using the disease–gene associations available in VitaGraph.
- **Candidate generation:** We generate a pool of candidate triples that are *not already present* in the graph. Two common strategies are applicable: (a) candidate compound–disease triples (when compound–disease relations are available for the disease of interest), and (b) candidate compound–gene triples, where genes are restricted to the hypertension-associated set. In both cases, the relation types can be restricted to interactions that are meaningful for intervention (e.g., TREATMENT/ACTIVATOR/BLOCKER, depending on the biological hypothesis).
- **Scoring and ranking:** The trained model is queried with the candidate triples to obtain a score for each one. Scores can be aggregated at the compound level (e.g., by taking the maximum score across the hypertension gene set, or by combining evidence across multiple genes/relations) to produce a ranked list of candidate compounds.
- **Filtering and re-ranking (optional):** To increase consistency and interpretability, additional logic can be applied. For example, candidates can be re-ranked by functional coherence with hypertension biology by exploiting the pathway information encoded in gene features: compounds whose (predicted) target genes overlap more strongly with hypertension-associated pathways can be prioritized.

From the final ranking, we selected the top 10 compounds (Table 12), including approved and experimental drugs as well as preclinical and research compounds. While clinical trials are needed to demonstrate the effectiveness of the repurposed drugs, we report evidence of their possible action on hypertension and blood pressure-relevant pathways and genes.

**Table 12:** Top 10 compounds described by their PubChem ID and name, along with their relationship with hypertension and blood pressure regulation, and literature references (*(7S–2-(2-aminopyrimidin-4-yl)-7-(2-fluoroethyl)-1,5,6,7-tetrahydro-4H-pyrrolo[3,2-c]pyridin-4-one).

PubChem ID	Compound Name	Relationship
10976469	Phosphonothreonine	MAPK1 inhibitor. MAPK signaling activation promotes structural changes in blood vessels, contributing to high blood pressure^73–76^
4592	Roxadustat	Blocks hypertensive responses in an Ang II model, preventing vascular thickening and cardiac hypertrophy^77^.
135418940	XAV939	Suppresses the Wnt/*β*-catenin signaling pathway, which is overactivated in hypertension^78–80^
16757525	*	Compound that inhibits the GSK-3 *β*, a key regulator in hypertension and vascular remodeling that contributes to proliferation of arterial smooth muscle cells and inflammation^81^.
67409219	Voruciclib	CDK9 inhibitor. CDK9 contributes to hypertension-related damage and is a potential cardiology drug target^82^.
71727581	Ravoxertinib	Inhibits the ERK pathway involved in vasoconstriction and vascular smooth muscle cell growth^83,84^
11338033	AT7519	Selective CDK inhibitor. Abnormal CDK activation promotes vascular smooth muscle proliferation in hypertension^85,86^
44462678	Phosphoaminophosphonic Acid-Adenylate Ester	Targets GSK-3 *β*, a key regulator in hypertension and vascular remodeling that contributes to proliferation of arterial smooth muscle cells and inflammation^81^.
3616	Hexamethylene Bisacetamide	Action on HEXIM1, which plays a protective role against right ventricular hypertrophy in pulmonary arterial hypertension models^87^. Moreover, this compound was found to prevent obesity, indirectly improving cardiovascular health^88^.
9863341	VTP-194204	Modulator of the retinoic acid receptor RXR *γ*. Activation of RXRs is protective against angiotensin II-induced hypertension and vascular remodeling^89^.

## Conclusion

In this work, we introduce VitaGraph, a knowledge graph specifically designed to support graph machine learning in biologically and chemically relevant applications. Building upon the DRKG dataset, we perform comprehensive data cleaning and node enrichment by incorporating biochemically meaningful features derived from diverse data sources. The resulting knowledge graph is intended to serve the research community in the discovery of novel biological insights, such as previously unidentified gene–gene interactions or drug repurposing opportunities, treated as link prediction problems. Our analysis demonstrates that relational graph neural network models can effectively learn from VitaGraph, indicating its suitability as a benchmark for graph machine learning and knowledge extraction models, and our drug repurposing use case shows its practical application in biomedical and pharmaceutical tasks. Moreover, the novel connections discovered through this framework, once experimentally validated, can be integrated into the graph, thereby continually enhancing the breadth and depth of the biomedical knowledge it contains. One limitation of our dataset lies in its reliance on the quality of the integrated data sources. Nevertheless, by maintaining regular updates, we aim to provide researchers with a reliable and continually improving resource for biomedical discovery.

## Data Availability

VitaGraph^66^ is freely available on Kaggle at: 10.34740/kaggle/ds/7415432.

## Code availability

The code comprising the pipeline to generate the dataset and the benchmarking experiments is freely available at: https://github.com/GiDeCarlo/VitaGraph.
